# Multiple Days of Heat Exposure on Firefighters’ Work Performance and Physiology

**DOI:** 10.1371/journal.pone.0136413

**Published:** 2015-09-17

**Authors:** Brianna Larsen, Rod Snow, Grace Vincent, Jacqueline Tran, Alexander Wolkow, Brad Aisbett

**Affiliations:** 1 Centre for Physical Activity and Nutrition Research (C-PAN), Deakin University, Melbourne, Australia; 2 Bushfire Co-Operative Research Centre, Melbourne, Australia; 3 Centre for Exercise and Sports Science, Deakin University, Melbourne, Australia; Bascom Palmer Eye Institute, University of Miami School of Medicine;, UNITED STATES

## Abstract

This study assessed the accumulated effect of ambient heat on the performance of, and physiological and perceptual responses to, intermittent, simulated wildfire fighting tasks over three consecutive days. Firefighters (n = 36) were matched and allocated to either the CON (19°C) or HOT (33°C) condition. They performed three days of intermittent, self-paced simulated firefighting work, interspersed with physiological testing. Task repetitions were counted (and converted to distance or area) to determine work performance. Participants were asked to rate their perceived exertion and thermal sensation after each task. Heart rate, core temperature (T_c_), and skin temperature (T_sk_) were recorded continuously throughout the simulation. Fluids were consumed ad libitum. Urine volume was measured throughout, and urine specific gravity (USG) analysed, to estimate hydration. All food and fluid consumption was recorded. There was no difference in work output between experimental conditions. However, significant variation in performance responses between individuals was observed. All measures of thermal stress were elevated in the HOT, with core and skin temperature reaching, on average, 0.24 ± 0.08°C and 2.81 ± 0.20°C higher than the CON group. Participants’ doubled their fluid intake in the HOT condition, and this was reflected in the USG scores, where the HOT participants reported significantly lower values. Heart rate was comparable between conditions at nearly all time points, however the peak heart rate reached each circuit was 7 ± 3% higher in the CON trial. Likewise, RPE was slightly elevated in the CON trial for the majority of tasks. Participants’ work output was comparable between the CON and HOT conditions, however the performance change over time varied significantly between individuals. It is likely that the increased fluid replacement in the heat, in concert with frequent rest breaks and task rotation, assisted with the regulation of physiological responses (e.g., heart rate, core temperature).

## Introduction

Wildfires can last hours, days, or weeks [1,2], depending on their severity. As a result, firefighters can be required to work long shifts (10 to 15 hours) over consecutive days [1,3–5], while performing a range of physically demanding tasks [6]. While some of these tasks involve the presence of a live fire, the majority of tasks performed during wildfire suppression are performed away from the fire, either during preparation for, or ‘mopping up’ after, a fire event [6]. In addition to the physical demand imposed by firefighting work [2,7], firefighters frequently perform their duties under a range of environmental conditions [1,8]. Australian firefighters performing wildfire suppression and recovery operations *after* a major fire event (the Victorian ‘Black Saturday’ Bushfires, 2009) have been observed to do so in peak ambient conditions ranging from 18.6–33.9°C [8]. During hot weather days, firefighters responding to a multi-day ‘campaign’ fire may also sleep under warm conditions, in temporary accommodation at (or near) the fireground [1,3]. Given this combination of potentially fatiguing factors, it is important for policy makers in the fire industry to understand the impact of performing consecutive work shifts in the heat. If firefighters cannot sustain their work performance over multiple work days it may have negative implications for the fire suppression effort as a whole. Slowed productivity may result in an increase in the time taken to control a wildfire, which may ultimately place firefighters, civilians, and their property at undue risk. Further, understanding the physiological and subjective responses to such work is important for fire agencies in preserving the health and safety of their personnel.

To date, there is a paucity of research examining the cumulative effect of heat exposure over multiple days, particularly in an occupational context. Research investigating construction workers performing three days of work under hot ambient conditions (ranging from 32.5 to 49.0°C) showed no difference in physiological variables (including urine specific gravity, aural temperature and heart rate), either within or between shifts [9]. The authors’ suggest that the workers were able to self-regulate their work output and fluid intake in order to avoid adverse physiological consequences (e.g., dehydration, heat stress); however, no measure of work performance was reported in this study. In contrast to these findings, sport acclimation research suggests, for the most part, that short-term repeated heat exposures will actually improve performance and decrease the thermal stress and exertion elicited during exercise [10–12].

Relying on the existing in-field or laboratory research to inform fireground workplace practices is problematic for several reasons. Laboratory studies that examine the relationship between heat and manual-handling performance have observed decrements in lifting, carrying, and pushing performance [13], and work tolerance time [14]. However, the short durations utilised (30 to 90 min) do not provide insight into the fatiguing effects of heat over longer work periods, or over consecutive days. The available multi-day heat acclimation studies typically use a pre-post design, where the focus is on performance adaptations that occur following a period of heat acclimation [11,12], rather than during. These heat-acclimation protocols also commonly use cycling [11,12] or rowing [10] as their measure of performance, and are most often restricted to 90 min (or less) of heat exposure per day. Wildland firefighters in the field perform intermittent-intensity [2,4,7], manual-handling work tasks such as digging, raking, carrying, and dragging [6], and work in shifts that last a minimum of 10 hours [1,3–5]. The exercise modes, intensities, durations, and work to rest ratios are so vastly different to that utilised in the heat acclimation protocols that it is difficult to extrapolate these findings to firefighters’ performance on the fireground. The existing field research more closely resembles the long-duration, manual-handling work profile of wildfire fighting, but does not report worker performance [9]. Thus, it is not possible to use this research to determine the effect that multiple days of heat exposure has on firefighters’ work output.

As such, this study aims to assess and describe the accumulated effect of ambient heat on the performance of repeated, long-duration simulated wildfire-fighting tasks over consecutive days. Further, this study aims to quantify the effect that consecutive days of heat exposure have on physiological and subjective responses during simulated firefighting work. Although multiple-day heat research is not yet developed enough to support a firm hypothesis, the current authors predict that performance of the simulated firefighting work will be negatively impacted by consecutive days of heat exposure. Specifically, that firefighters will decrease their rate of work on each task in the heat when compared to a temperate control condition, and that work output will be further reduced on the second and third days of heat exposure when compared to the first day. Secondly, it is predicted that this decrease in work productivity will regulate physiological and subjective responses so that they remain comparable to that observed in a more temperate environment.

## Materials and Methods

### Participants

Male and female volunteer and career firefighters (n = 36) participated in this study (~14% female, in accordance with the ratios observed within Australia’s firefighting population [15]). Participants in each group were matched (in order of priority) by age, gender, and body mass index [16] in order to minimise variation between groups. Participants were also matched according to the sequence in which they completed the physical work circuit (see *Experimental Protocol* for details). A self-report measure of habitual physical activity was also collected from each participant in order to glean an indication of their aerobic fitness levels, given this is a factor known to influence exercise performance in the heat. Unfortunately direct measures of aerobic fitness were unable to be conducted due to time and cost constraints. Firefighters from hotter climates (e.g., Northern Australia) were excluded from the study in order to control a potentially confounding variable (heat acclimation), and reduce variation in the participant cohort. Data collection also took place during the autumn and winter months to minimise the effect of heat acclimation. Participants provided written informed consent and filled out a Medical Questionnaire prior to commencement of the study to ensure they were physically able to perform the work protocol. Ethical approval was obtained for this study by Deakin University (DUHREC 2014–040).

Prior to testing, participants’ height was measured and recorded using a stadiometer (Fitness Assist, England), and semi-nude body mass was measured using an electronic scale (A and D, Japan). In all trials, participants wore firefighting personal protective clothing (PPC). This included a two-piece jacket and trouser set made from Proban cotton fabric (Protex, Australia), suspenders, boots, gloves, and helmet (amounting to ~5 kg), which meet the performance requirements for wildland firefighting clothing as stipulated by the International Organization for Standardization (ISO 15384:2003).

### Experimental Protocol

Participants were allocated to either a control (CON; 19°C, 58% RH) or hot (HOT; 33°C, 40% RH) environmental condition to reflect the different ambient environments often faced on the fireground [8], and were tested in groups of five (or less). Ambient temperature was maintained through the use of split cycle air conditioners (Panasonic, Japan) and portable ceramic disk heaters (Micro Furnace, Sunbeam, Australia), and was continuously measured and logged using three temperature nodes (Onset Computer Corporation, USA). Participants were required to perform three days of simulated fire suppression work, including physical work as well as physiological and cognitive tests. The suite of cognitive tests was part of a broader study; thus, the methods surrounding these protocols and the ensuing results will not be described.

Testing took place in a windowless, climate-controlled room measuring 9 × 13 m. The distance between the various work stations was precisely measured during each testing session to ensure a consistent standard was being met between groups. Participants were familiarized with the physical work tasks, as well as the rating of perceived exertion (RPE) [17] and thermal sensation [18] scales, on the day prior to the commencement of testing. Participants were also then made aware of the condition to which they’d been allocated (CON or HOT). Participants ingested a core temperature capsule (Jonah, Minimitter, Oregon) prior to sleep (9:30 pm) to allow adequate time for the capsule to pass through the stomach, thereby preventing inaccurate readings occurring as a result of ingested food or liquid [19]. Core temperature pills were administered each consecutive night throughout the protocol, in order to capture accurate core temperature readings during the following day of simulated work.

Prior to the commencement of testing each day, participants had heart rate monitors (Polar, Finland) and skin temperature patches (VitalSense/Jonah, Minimitter, Oregon) affixed. Core and skin temperature recorded continuously on a data logger (VitalSense, Minimitter, Oregon); therefore ‘pre-work’ readings were extracted from the data post-testing. Participants then dressed in their firefighting PPC, and began testing at 12:30 pm (on day one) in both conditions.

On day one, participants performed three work ‘circuits’, each lasting two hours (six-hour total work period), and comprising 55 min of physical work, 20–25 min of physiological data collection, 20–25 min of cognitive testing and a 15–20 min rest period. On days two and three, participants performed five of the two-hour testing circuits (10-hour work periods) to simulate the often-long work shifts performed during a campaign fire deployment [1,3–5]. Thus, over the thee-day simulation all firefighters performed 13 work circuits in total. Participants were allowed a half-hour lunch break in the middle of the workday (on days two and three).

The work to rest ratios that made up each two-hour circuit mimic the ratios of actual fireground work [4,5,7], and the physical tasks were designed to simulate the movements and fitness components of fire suppression tasks in the field [6,20]. Each participant had the same work opportunity each circuit; however the starting position (task) was staggered to allow multiple participants to be tested at once. Once allocated to a circuit order, participants followed the same sequence in all 13 work circuits.

Participants were allowed to drink room temperature water ad libitum throughout testing. Prior consultation with fire agencies determined that participants were also supplied with sachets of flavoured electrolyte supplement, and a ‘ration pack’ of snack foods (e.g., muesli bars, crackers, and confectionary) similar to that which they would receive on the fireground. Food and drink consumption was only permitted during the rest periods between tasks, not during the performance of the physical work tasks.

#### Physical Work Circuit

The work circuit comprised six physical tasks, which were chosen on the basis of being the most physically demanding tasks performed by Australian rural firefighters [6]. These tasks are also considered the most important tasks in achieving operational outcomes [6], and have been shown to be the most frequent, and/or longest and most intense, tasks performed during wildfire suppression work [20]. All hose tasks employed in the protocol were performed using 38-mm hoses with branch attached. The tasks included:

*Rakehoe work*—involved raking 29-kg of material (large and small tyre crumb) from one end of a rectangular wooden box (2 × 0.9 m) to the other, using a rakehoe, to simulate building a firebreak [6,21].

*Blackout hose work*–involved walking the perimeter of a 2.5- × 2.5-m square, stopping at each corner for three seconds (as timed by a metronome). Whilst walking, participants dragged a 15-kg weight attached to a 2-m hose. This task simulates the ‘stop-start’ dragging of a charged hose when dousing smouldering debris with water during post-fire clean up work [6].

*Hose rolling*–a common task performed during pack up, this task required participants to roll up a 16-m hose (folded in half to a length of 8 m) to an operational standard [6].

*Lateral hose repositioning*–involved walking in an arc (3.5-m radius; 11-m length), carrying a 3.5-m hose. The hose was attached to a weighted stand centred at the base of the arc. Two platforms (68 × 28 × 15 cm; Spalding, Australia) served as ‘obstacles’ (e.g., logs, fallen debris), that participants had to manoeuvre. This task simulated moving a charged fire hose sideways from a fixed point, such as a water source [6].

*Charged hose advance*–simulated walking forwards with a hose filled with pressurised liquid [6]. Participants dragged a 15-kg weighted tyre attached to a 2-m hose, up and down a marked distance of 8 m.

*Static hose hold*–involved pointing a 3.5-m hose (attached to a weighted stand via an elastic strap, to provide resistance) at a target (with laser pointer attached). Participants were instructed to hold the laser within the target for five minutes, or until exhaustion. If the laser moved out of the target, or if the hose touched the ground, for more than two seconds, participants’ performance time was recorded at that point. This simulated holding a charged hose in position for an extended period when dousing a fire [6]. All participants in both conditions were able to complete the maximum time of five minutes, so performance results for this task will not be reported.

Five min was allocated to each task (inclusive of the time taken to move from one station to the next); however, the work to rest ratios within that time varied between tasks, according to the work to rest ratios observed for each task in the field (Table 1) [20]. Similarly, some tasks were performed only once in each 55-min physical work period, whereas others were performed multiple times according to their frequency in the field (Table 1) [20]. While the work and rest periods were standardised across groups, firefighters could self-select their work output within the work periods (e.g., by increasing or decreasing the number of repetitions performed in the given time frame). The repetitions completed during each work task were recorded through the use of a specially designed iPad application (Good Dog Design, Australia). In analysing the data, repetitions were converted into distance (m), or area (m^2^) in the case of the rakehoe task. Each of the physical work tasks was analysed individually, based on the amount of work that was performed at each station during each of the physical work circuits (circuits 1–13). For brevity, daily mean performance data will be presented in the Results.

**Table 1:** Task frequencies and work to rest ratios.

Task name	Work to rest ratio	Times performed each circuit
Rakehoe work	90 s work: 60 s rest: 90 s work	1
Blackout hose work	90 s work: 60 s rest: 90 s work	2
Hose rolling	60 s work: 60 s rest: 60 s work	1
Lateral hose repositioning	30 s work: 30 s rest × 4	4
Charged hose advance	65 s work: 55 s rest: 65 s work	1
Static hose hold	5 minutes continuous work (maximum)	1
Dedicated rest break	5 minutes	1

#### Overnight Protocol

Over the three days of testing, participants ‘lived’ in the simulated environment at the testing facility, and adhered to a strict schedule throughout their stay. This included set meal, sleep, and physical, physiological, and cognitive testing times throughout each day. Caffeine and cigarettes was permitted; however participants were encouraged to consume them only as they would during a multi-day campaign fire, and only during rest periods (i.e., not during physical or physiological testing). All food and drink was provided to participants, based on the standard food items that would be available to firefighters on the fireground (according to consultation with industry). This included a hot breakfast (bacon, eggs, baked beans, and toast), sandwiches for lunch, and a hot dinner (e.g., pasta, meat, and vegetables).

Testing concluded at 6:30 pm on each of the three testing days. On nights one and two, participants then ate dinner in the testing environment (on the third night 6:30 pm represented the conclusion of testing). After dinner, participants left the testing environment briefly to shower and prepare for bed. Once showered and dressed for bed, firefighters had approximately 2.5 hours of ‘free time’ (within the testing environment), where they were permitted to watch movies, read, or play card/board games. Participants were in bed by 9:45 pm, with lights out at 10:00 pm. Overnight temperature was maintained at 18°C during the control trial, and 23°C during the hot condition. This simulated the often warm sleeping environment firefighters experience on a campaign tour [1,3].

Participants were woken at 6:00 am on days two and three, where they dressed and prepared for the day. After breakfast, participants were again fitted with a heart rate monitor and skin temperature patches, and baseline physiological measures were recorded. During this time, the temperature was increased to 33°C in time to commence the ‘workday’ at 8:00 am.

#### Physiological and Subjective Measurements

Core temperature, skin temperature, and heart rate were recorded continuously throughout the three simulated work ‘shifts’. Maximum heart rate (HR_max)_ was predicted using the formula 207–0.7 × age [22] in order to account for the substantial variation in participant age (range: 18–60 years). Skin temperature was recorded at four sites on the left side of the body; the middle of the chest, thigh, upper arm and calf [23]. Mean skin temperature was calculated using the formula 0.3(t_chest_ + t_arm_) + 0.2(t_thigh_ + t_leg_) [24]. The types and quantities of ingested food and liquid were recorded throughout testing, and food and drink data was extracted using the FoodWorks 7 nutrition software (Xyris Software Pty Ltd, Australia). All urine was measured for volume, and USG was analysed using a portable refractometer (Atago, Japan), to determine hydration status. Given that day one was shorter than days two and three, all fluids ingested and urine expelled were expressed relative to the number of circuits each day. Finally, participants were asked to provide RPE [17] and thermal sensation [18] ratings after each individual physical task (i.e., every five minutes).

#### Statistical Analysis

Statistical analyses were carried out using the IBM Statistical Package for the Social Sciences (SPSS V.22.0.0, Champaign, Illinois) and Stata 12.0 (StataCorp, Texas, USA). The distribution of the data was tested for normality using visual inspection of Q-Q plots, formal testing (Shapiro Wilk tests), and by calculating skewness and kurtosis [25]. All variables met assumptions of normality with the exception of ingested and expelled fluids, as 1) Q-Q plots deviated substantially from the line of identity, 2) the Shapiro Wilk tests were significant (P < 0.05), and 3) they fell outside the acceptable range for kurtosis values (kurtosis > 2 or < -2) [26]. Participant characteristics, ambient temperature, relative humidity, energy, smoking status, and caffeine consumed were analysed using t-tests to determine differences between the two experimental conditions. For all other variables, the Generalized Linear Latent and Mixed Model approach was used (*gllamm* software, version 2.3.20) [27,28].

The GLMM modelling procedure more accurately accounts for the serial correlation of data points over time, and is therefore increasingly preferred over repeated-measures ANOVA for these types of data sets [29]. This generalized technique allows for non-normal distributions to be specified if the data does not meet the assumption of normality [30]. Thus, models were constructed with a gamma distribution for the ingested and expelled fluid variables, but did not converge. Therefore, the authors proceeded to use models with a normal distribution. The implications of fitting normal models to non-normally distributed data will be further discussed in the Discussion section of the manuscript. The *gllamm* software also provides valid estimates in the presence of missing data, and uses maximum likelihood estimation with adaptive quadrature for more reliable parameter estimates than quadrature [27]. In the current study, < 2% of performance, heart rate, perceptual, and hydration data was missing. For core and skin temperature variables, 20% of the data was missing due to device malfunction, and thus was treated as missing-at-random. Mixed effects models incorporate fixed effects to assess the influence of the experimental intervention, as well as random effects that factor in the unique responses of individuals (e.g., individual differences in response to heat exposure) [31]. Thus, mixed models are able to differentiate between-subject from within-subject effects. The construction of the mixed models followed the iterative framework recommended by Singer [32], where the fixed effects of Condition (categorical variable), Circuit (continuous variable), and Condition × Circuit were analysed, with random intercepts and random slopes that varied at the Participant level. Given that the circuits were performed with only brief breaks in between, it is likely that the physiological responses and/or physical fatigue accrued during one circuit would impact the following circuit (e.g., if core body temperature was increased after the first circuit of work, it is possible that values would not return to baseline levels before the beginning of the subsequent circuit). Therefore, performance and physiological responses were treated as time-series data because of the likely serial dependency between consecutive circuits. In this way, GLMMs allow rates of change to be assessed, rather than conceptualising time points as discrete categories of time (as done by ANOVAs with post-hoc tests) [33,34].

As all dependent variables were treated as having a Gaussian (e.g., normal) distribution, the ‘identity’ link function was specified [35]. The CON and HOT groups were coded 1 and 0, respectively. Therefore, a positive beta (β) value for the effect of Condition indicates that HOT > CON, and a negative β value indicates that CON > HOT. For Circuit (time) effects, a positive β value indicates an increase over time (e.g., over successive circuits), and a negative β value indicates a decrease. Thus, when using the GLMM method, parameter estimates (presented as β values) reflect the magnitude of the difference between groups (or over time). The random effects explain the variance due to inter-individual differences at baseline (random intercept) and over time (random slopes). In selecting the optimal model for each variable, Akaike weights (which determine the relative likelihood of the model, given the data) were compared between models as per the procedure outlined in Burnham and Anderson [36]. The final parameter estimates for all GLMM variables are reported as β coefficient ± standard error of the estimate (SE). Statistical significance was set at P ≤ 0.05, and all other data are presented as means ± standard deviations.

## Results

There was no difference between the CON and HOT groups for participants’ age, height, weight, BMI, the ratio of males to females, self-reported habitual caffeine consumption, or physical activity (P ≥ 0.481; Table 2). Two participants in the CON group were smokers, whereas all participants in the HOT group were non-smokers (P = 0.154). There was also no difference (P = 0.605) in the amount of energy consumed each day between the CON (14,706 ± 3774 kJ) and HOT (15,080 ± 3489 kJ) groups. There was, however, a significant difference (P = 0.019) in the amount of caffeine consumed each day during the simulation. The CON and HOT groups consumed on average 80 ± 75 and 121 ± 97 mg per day, respectively. Average room temperature and relative humidity (%) during the simulated shifts were also different between conditions (P < 0.001), reaching 32.50 ± 1.30°C and 39.56 ± 2.80% in the HOT group compared to 19.16 ± 0.25°C and 58.26 ± 2.78% in the CON group.

**Table 2:** Characteristics of firefighters in the CON and HOT conditions. All data are reported as means ± SD.

	CON	HOT
**n**	18	18
**Age (years)**	39 ± 16	36 ± 13
**Height (cm)**	178 ± 8	178 ± 9
**Weight (kg)**	84.9 ± 17.8	88.0 ± 18.0
**BMI (kg.m** ^**-2**^ **)**	26.7 ± 4.9	27.5 ± 3.5
**Males: Females**	15: 3	14: 4
**Habitual daily caffeine intake (mg)**	183 ± 126	196 ± 130
**Habitual physical activity (sessions per week)**	3.2 ± 3.0	3.2 ± 2.4

### Work performance

Raw data for all task-based variables, including daily mean performance (per circuit), are presented for ease of comparison between conditions (Table 3). All participants completed the 5-minute maximum static hose hold, thus performance is not reported for this task. For all of the physical work tasks, the fixed effect of Condition did not explain a significant amount of variance in work performance (P ≥ 0.321). Random slope models best explained the variance in work performance for all tasks (P < 0.001), highlighting the individual variability in both an individual’s starting point and their performance responses over time.

**Table 3:** Daily mean work performance, heart rate, RPE and thermal sensation data across the CON and HOT conditions. Work performance is reported as distance (m) for all tasks except rakehoe work, which is reported in area (m^2^). All data are reported as means ± SD.

			Work performance			Heart Rate (% HR_max_)			RPE			Thermal sensation		
			Day			Day			Day			Day		
TASK			1	2	3	1	2	3	1	2	3	1	2	3
**Blackout hose**	CON	Mean	165.5	168.7	166.8	65	62	62	12.1	11.9	12.1	4.9	4.7	4.8
		SD	12.7	12.8	12.8	12	10	10	0.9	0.8	0.9	0.4	0.6	0.5
	HOT	Mean	180.2	171.6	175.0	68	61	60	12.0	11.5	11.5	5.6	5.5	5.6
		SD	27.8	23.0	22.6	10	8	7	1.0	0.9	0.8	0.6	0.5	0.5
**Charged hose advance**	CON	Mean	104.9	111.2	116.6	77	74	73	15.5	15.4	16.1	5.6	5.5	5.5
		SD	24.2	24.0	28.7	13	14	13	1.4	1.3	1.7	1.0	1.0	1.1
	HOT	Mean	106.0	112.1	114.5	76	71	70	14.7	14.6	14.5	6.2	6.0	6.0
		SD	25.9	32.7	27.0	10	9	7	1.6	1.5	1.4	0.6	0.7	0.7
**Lateral hose repositioning**	CON	Mean	617.2	656.8	688.0	65	63	63	11.3	11.3	11.5	4.7	4.6	4.7
		SD	91.1	83.1	86.1	11	10	10	0.7	0.8	0.9	0.5	0.6	0.5
	HOT	Mean	646.5	628.9	624.2	66	60	59	11.0	10.7	10.7	5.5	5.4	5.4
		SD	103.7	97.8	104.9	9	8	7	1.1	1.3	1.1	0.5	0.4	0.4
**Hose rolling**	CON	Mean	17.9	21.6	24.6	66	65	64	12.3	12.3	12.8	4.8	4.7	4.8
		SD	4.1	5.0	7.1	11	10	10	1.1	1.2	1.5	0.6	0.7	0.7
	HOT	Mean	17.9	21.5	23.8	67	63	61	11.0	11.3	11.6	5.3	5.4	5.5
		SD	6.1	8.0	7.9	11	10	8	0.9	0.5	0.8	0.5	0.5	0.4
**Rakehoe work**	CON	Mean	4.9	5.4	5.6	73	71	70	14.6	14.5	14.9	5.7	5.5	5.7
		SD	1.3	1.4	1.4	10	8	9	0.9	1.3	1.3	0.6	0.7	0.7
	HOT	Mean	4.9	5.3	5.7	76	69	67	14.3	14	14.1	5.9	6.0	6.1
		SD	1.1	1.1	1.3	10	8	8	1.7	1.4	1.8	0.7	0.6	0.6
**Static hose hold**	CON	Mean	-	-	-	65	60	59	13.7	13.1	13.2	5.4	5.3	5.5
		SD	-	-	-	13	11	11	2.0	1.9	1.8	0.9	0.9	0.7
	HOT	Mean	-	-	-	69	61	59	12.7	12.0	11.5	6.1	6.0	5.8
		SD	-	-	-	11	8	7	1.7	1.4	1.3	0.6	0.7	0.7

### Core and skin temperature

**Fig 1:**
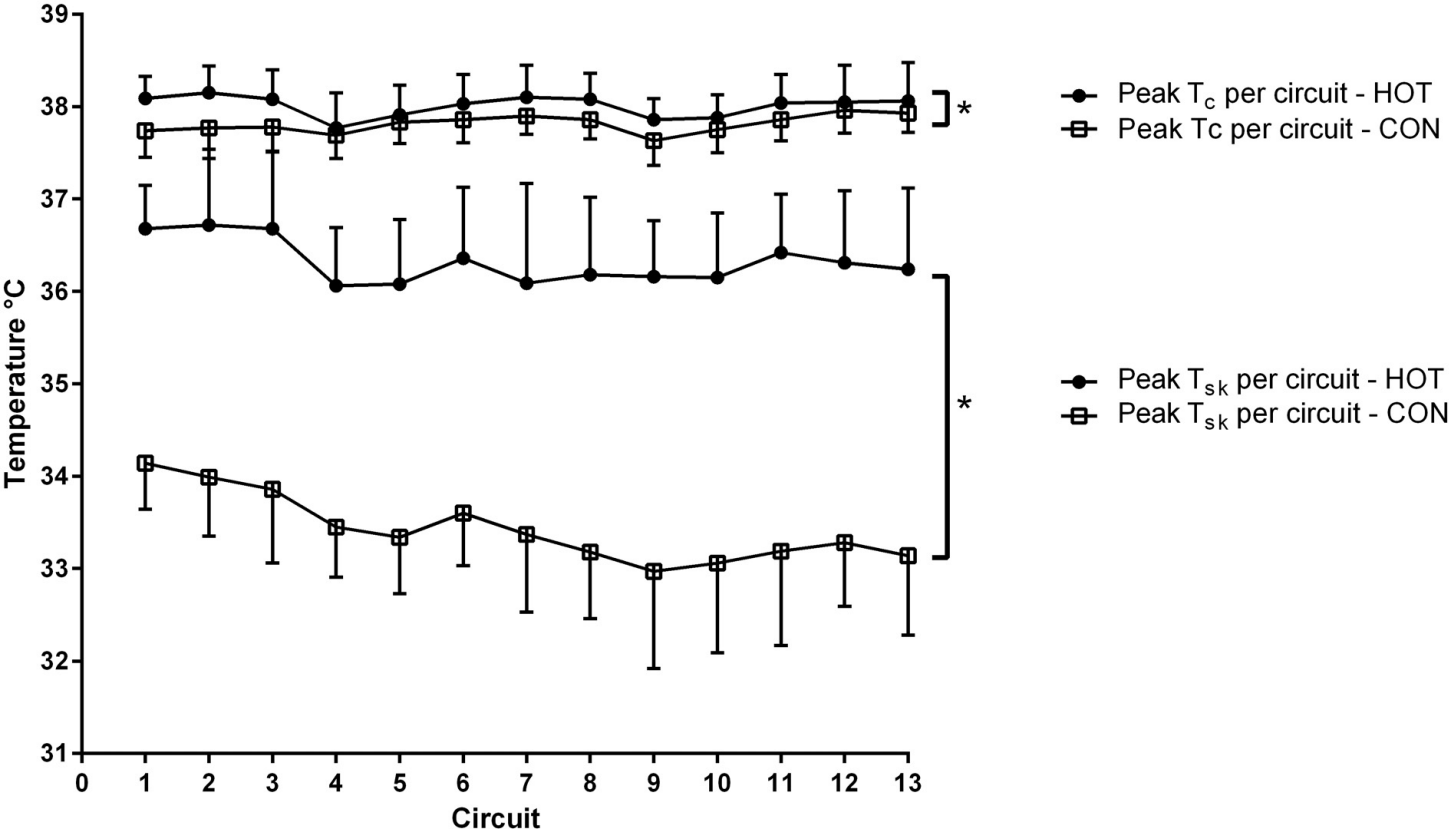
Peak core and skin temperature over the 13 work circuits. All data are presented as means ± SD. T_c_ = core temperature, T_sk_ = mean skin temperature. * indicates that HOT significantly higher (P < 0.05) than CON.

The variance in average core and mean skin temperature each circuit was best explained by the full model. For mean skin temperature there was a Condition × Circuit effect, which showed that participants in the HOT condition were hotter, and that this difference increased each successive circuit (β = 0.03 ± 0.01°C; P = 0.014). Both core and mean skin temperature also displayed significant fixed effects for Condition (β = 0.30 ± 0.08 and 3.07 ± 0.27, respectively; P < 0.001). The conditional growth model with random slopes explained the greatest amount of variance in peak core and mean skin temperature per circuit. As such, peak core and mean skin temperature were significantly higher in the HOT when compared to the CON condition (P ≤ 0.002), reaching on average 0.24 ± 0.08°C and 2.81 ± 0.20°C higher, respectively (Fig 1).

### Heart rate

Daily mean heart rate data per work task is presented in Table 3. For all physical work tasks, the fixed effect of Condition did not explain a significant amount of the variance in firefighters’ heart rate (P ≥ 0.477). The variance in heart rate across all tasks was best explained by the random slope models (P < 0.001), highlighting a large amount of individual variation. Similarly, there was no effect of Condition on average heart rate during the 2-hour circuits (P = 0.986), or when the 55-minute work circuits (P = 0.717) or 65-minute rest periods (P = 0.660) were analysed individually (data presented in Table 4). Again, random slope models best explained the variance in heart rate during each of these periods (P < 0.001). Conversely, the peak heart rate reached each circuit was best explained by the conditional growth model with random slopes (β = -6.79 ± 3.11; P = 0.029), with a fixed effect indicating that participants in the CON condition reached a higher peak heart rate when compared to their HOT trial counterparts.

**Table 4:** Daily heart rate data across the CON and HOT conditions. Heart rate data are expressed as a percentage of age-predicted maximum (% HR_max_). All data are reported as means ± SD.

	Mean heart rate per circuit (% HR_max_)			Peak heart rate per circuit (% HR_max_)			Mean heart rate per 55-minute work bout (%HR_max_)			Mean heart rate per 65-minute rest period (%HR_max_)		
	Day			Day			Day			Day		
	1	2	3	1	2	3	1	2	3	1	2	3
**CON**	59 ± 10	56 ± 8	56 ± 8	91 ± 11	87 ± 11	88 ± 11	66 ± 11	63 ± 9	63 ± 10	52 ± 9	50 ± 8	49 ± 7
**HOT**	61 ± 9	56 ± 7	54 ± 6	87 ± 10	82 ± 9	82 ± 7	68 ± 9	62 ± 8	60 ± 7	55 ± 8	50 ± 7	49 ± 6

### Perceptual responses

All mean daily RPE and thermal sensation data are presented in Table 3. The conditional growth model with random slopes best explained the variance in thermal sensation for the blackout hose, charged hose advance, hose rolling, and static hose hold tasks. For these tasks, thermal sensation was significantly higher in the HOT trial, despite significant variations in individual response (P ≤ 0.029). For the lateral hose repositioning task, the conditional growth model best explained the variance in thermal sensation (β = 0.78 ± 0.15; P < 0.001), again indicating that on average firefighters felt hotter when performing this task in the HOT trial. Alternatively, the Circuit (time) only model best explained the variance in thermal sensation responses during the rakehoe task (β = 0.02 ± 0.01; P < 0.016), which demonstrates that participants reported higher thermal sensation values as the trial progressed, irrespective of condition. For firefighters’ RPE scores, random slope models explained the most amount of variance for the raking and lateral hose repositioning tasks (P < 0.001). However, for the blackout hose (β = -0.53 ± 0.16; P = 0.001), charged hose advance (β = -0.95 ± 0.45; P = 0.033), static hose hold (β = -1.34 ± 0.53; P = 0.012), and hose rolling (β = -1.19 ± 0.28; P < 0.001) tasks, conditional growth models with random slopes reported main effects of Condition, highlighting that participants rated these tasks as more exerting in the CON trial.

### Hydration

**Fig 2:**
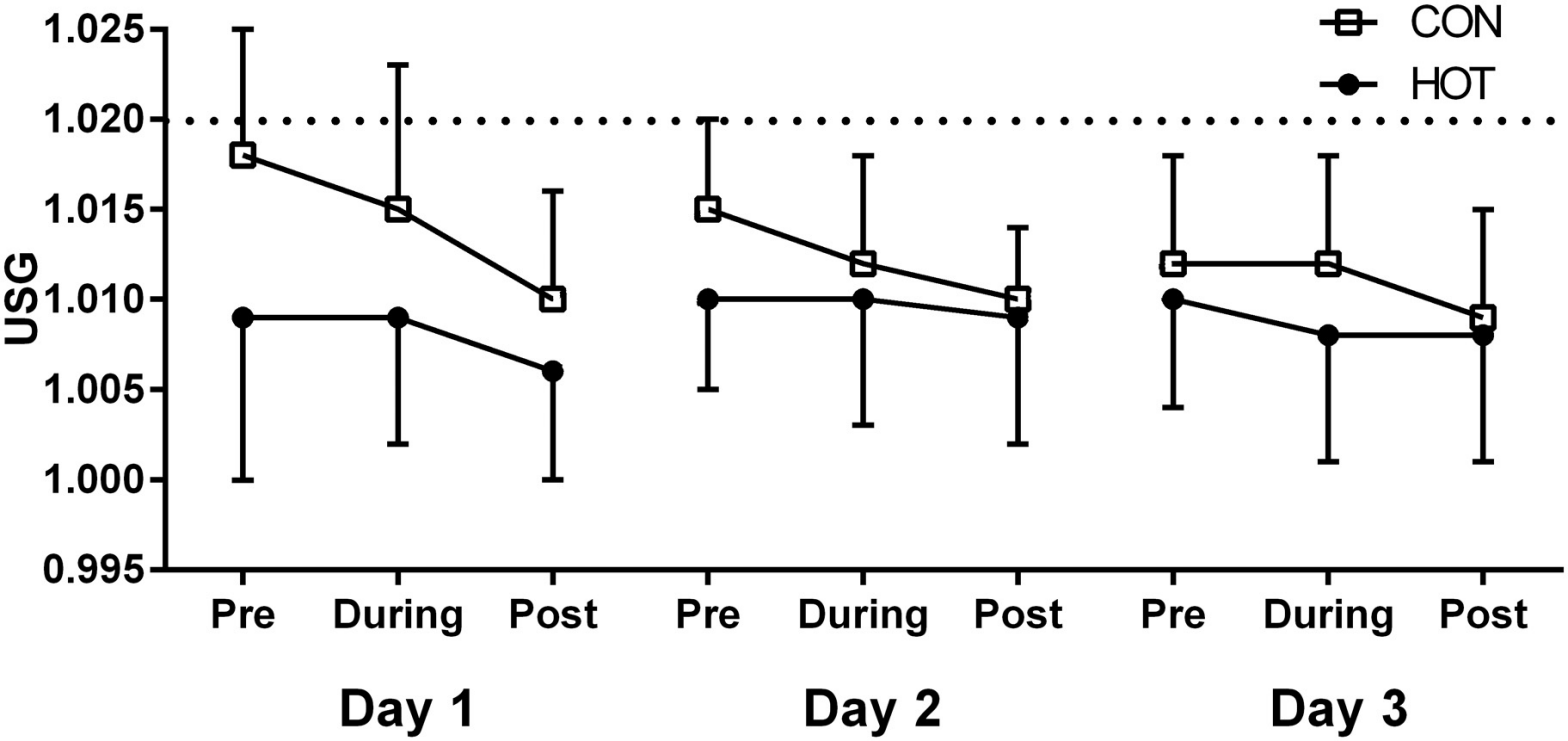
Pre, during, and post-shift USG values over the three days. * The dotted line denotes that the threshold for dehydration is > 1.020.

The variance in daily fluid consumption was best explained by the full model, with significant fixed effects for Condition (β = 930.91 ± 207.57; P < 0.001). Firefighters in the CON trial consumed 1175 ± 412 mL, 917 ± 242 mL, and 831 ± 287 mL per circuit on days one, two, and three, compared to 2141 ± 838 mL, 1501 ± 354 mL, and 1660 ± 432 mL in the HOT condition. The full model also best explained firefighters’ USG values, with significant effects of Condition (β = -0.007 ± 0.002; P < 0.001) and Circuit (β = -0.001 ± 0.000; P < 0.001; Fig 2). The variance in urine output volume across all tasks was best explained by the random slope models (P < 0.001), indicating that there was a high level of individual variation. Average urine volume per circuit (across the three days) was 604 ± 283 mL in the CON, compared to 829 ± 419 mL in the HOT.

## Discussion

Contrary to the authors’ predictions, work performance across all physical work tasks was unaffected by the heat. Rather, it was observed that, irrespective of experimental condition, initial performance was different between individuals, and the time course of performance change was also individual-specific. The secondary prediction, that physiological and subjective responses would be moderated by work output, and thus, comparable between conditions, was also not supported by the findings. While there was little difference in heart rate values between groups, all measures of thermal stress (core temperature, skin temperature, and thermal sensation) were elevated in the HOT condition. Conversely, USG values were significantly elevated during the CON trial, as were RPE scores for the majority of work tasks.

Despite its occupational relevance, there is a distinct lack of research investigating the effect of heat on work performance over consecutive days. Thus, in predicting performance outcomes in the present study the authors had to draw upon the (albeit limited) research that has examined the relationship between heat and manual-handling performance over short durations. In these studies, manual-handling work performance was decreased in hot relative to more temperate ambient conditions [13,14], a finding that was not replicated in the present study. Random slopes models best explained the variation in the current data set, highlighting the significant individual variability in performance responses over time. In other words, irrespective of experimental condition, some individuals improved their performance, some maintained performance, and some recorded performance decrements. It is well established that a multitude of factors determine how an individual will respond to exercise heat stress (e.g., gender, body composition, age, acclimation status) [37,38]. While the current data set is underpowered to allow for the inclusion of more factors into the model, assessing the contribution of individual characteristics and how they impact physical performance in this population warrants further research. In a practical setting, this would allow fire agencies to tailor work strategies according to individual characteristics that moderate the influence of heat on firefighters’ physical performance. Nevertheless, at a group level, the present findings suggest that firefighters are able to perform similarly in both temperate and moderately hot conditions. This is an encouraging finding for fire agencies, as it indicates that firefighters can be equally effective (in terms of their fire suppression efforts) under varying environmental conditions. However, whether this finding would persist in even hotter ambient environments, as occasionally encountered during wildfire suppression [39], remains unknown. Likewise, it is possible that firefighters performing single tasks extending over longer durations (e.g., > 5 minutes) under hot conditions may eventually succumb to physical fatigue.

Firefighters in the HOT condition experienced greater increases than the CON group across all measures of thermal stress (core temperature, skin temperature, and thermal sensation). Previously, construction workers performing three days of work under hot-very hot ambient conditions have been observed to maintain their physiological responses (including aural temperature), both within and between shifts [9]. However, no control group was used in this study. Thus, it is highly possible that, even though the workers’ physiological responses were relatively stable over a three-day period, they may have been significantly elevated relative to workers in a more temperature environment (as in the present research). It should be noted that, although the difference in peak skin temperature between groups in the current study was relatively substantial (2.81 ± 0.20°C), peak core temperature in the HOT condition was only 0.24 ± 0.08°C higher per circuit than in the CON trial, and was consistently < 38.20°C (see Fig 1). Therefore, while this difference between groups was statistically significant, it was compensable and may not translate to a meaningful effect on the fireground. It is possible that the regular rest periods utilised throughout the protocol, as well as adaptive behaviours (e.g., increased fluid intake, jacket and helmet removal during rest breaks), allowed participants to maintain manageable core temperature values across the course of the protocol.

Firefighters in the HOT condition almost doubled their fluid consumption (per circuit) relative to the CON group over the course of the three-day simulation. However, fluid consumption results must be interpreted with caution, given that models with a normal distribution were fitted to the data (which was not normally distributed) due to a lack of convergence when using gamma models. This particular data set was leptokurtic (kurtosis > +2), which means that the curve of the data displayed a higher peak and fatter tails than a normal distribution [40]. Thus, the parameters of the models are likely to underestimate true probabilities about the mean, and at extreme values [40]. In the absence of skewness, data with a leptokurtic curve will still provide valid parameter estimates (i.e., mean values), however the variance in the data may be biased (i.e., SE may be under- or over- estimated) [41]. The difference in mean fluid consumption between groups was reflected in firefighters USG findings, in which the HOT group recorded consistently lower values than their CON trial counterparts (see Fig 2) from the beginning of the three-day protocol. This difference in pre-shift USG values was evident even on day one, which indicates that participants in the HOT group had an anticipatory increase in fluid consumption leading into the protocol (e.g., the night prior). While it must be noted that both HOT and CON groups were classified as ‘hydrated’ throughout the simulation [42,43], the observed difference in both fluid consumption and USG values likely played a role in moderating all physiological variables in the heat. Previous heat research has shown that adequate fluid replacement during exercise assists with moderating heat-induced increases in core temperature [44], ratings of perceived exertion [44,45], and minimises cardiovascular drift [38,45,46]. In the current study, firefighters’ heart rate was comparable at all times with the exception of the peak heart rate achieved each circuit, which was higher in the CON group. Similarly, RPE values were higher in the CON group for four of the six physical work tasks. It is likely, then, that the increased fluid replacement observed in the HOT condition played a role in blunting both heart rate and RPE responses. There is also a small possibility that the elevated caffeine consumption in the heat (relative to the CON group) dampened firefighters’ perception of exertion [47]. However, firefighters in both groups consumed less caffeine during the protocol than their self-reported daily habitual caffeine consumption. Given this, and that the amount of caffeine consumed fell well below the 3 mg.kg^-1^ commonly associated with exercise benefits [48], it is unlikely that caffeine played an influential role on RPE or work performance in the current research. Irrespective of cause, the average difference in RPE between conditions across all tasks (and days) was 0.8 units. On a 6–20 point scale, a difference of this magnitude is unlikely to transfer itself to a meaningful difference during actual fire suppression.

There is a small possibility that intra-group differences (e.g., cardiorespiratory fitness) may also partially explain the observed RPE and heart rate findings. Although firefighters across the two experimental groups were matched for age, gender, and BMI, and there was no difference in participants’ self-report physical activity (in sessions per week), no objective measure of ‘fitness’ was utilised when matching participants. Therefore, it is possible that some of the firefighters allocated to the HOT condition had higher cardiorespiratory fitness levels relative to their CON group counterparts, and thus found the work less exerting. Nevertheless, the generalized mixed models used in the analysis factored in the unique responses of individuals to an intervention [31], and thus, should have been robust enough to account for the effects of individual subject variability. It is, however, important to note that the study design may have some other limitations that prevent direct extrapolation of the results to the fireground. Firstly, while conducting a laboratory study allowed for the accurate quantification of performance, physiological, and perceptual responses during heat exposure, it is likely that the artificial environment was not entirely representative of fire suppression in the field. Care was taken to ensure that the protocol ‘mimicked’ a campaign fire environment wherever possible (e.g., in the physical work tasks chosen, the food provided, types of bedding used etc.). However, the variability of an outdoor environment (e.g., changes in wind speed and direction) and the urgency of certain aspects of fire suppression were unable to be captured in a simulated setting. Further, radiant heat was not simulated in the present study design. However, it has been observed that radiant heat (from the sun or fire) accounts for very little of the total heat load placed on firefighters, particularly during tasks performed away from the fire [49]. Given that the majority of tasks in the present study protocol simulated either preparatory or post-fire ‘clean up’ work, replicating radiant heat load was not considered a primary objective of this research. However, segregating the effects of radiant heat from air temperature may be an avenue for future research. Lastly, while the physical work tasks used in the protocol were highly representative of actual fire suppression work (based on both field research and exhaustive consultation with industry experts), it is possible that the tasks were not sensitive enough to detect small changes in performance during heat exposure. Task validity was prioritised over reliability in the current research in order to maximise the transferability of results to the fireground. Therefore, while there is a possibility that task variability may have influenced the data, it is not likely that any undetected changes in performance would translate into a measureable performance difference in the field.

## Conclusions

There was no difference in firefighters’ work output between the CON and HOT conditions. Rather, performance responses over time varied significantly from individual to individual. While all measures of thermal stress were significantly elevated in the HOT trial, the heat load was compensable in both conditions, and the small (though significant) difference in core temperature between experimental groups would unlikely lead to any adverse health outcomes in an applied setting. Firefighters doubled their fluid consumption in the HOT trial, and thus recorded significantly lower USG values relative to the CON group (though both groups fell within the ‘hydrated’ range). It is likely that this increased fluid replacement in the heat, in concert with the frequent rest breaks and task rotation employed in the study, assisted with the regulation of physiological responses. These findings are promising from a fire agency perspective; not only were firefighters observed to be equally as operationally effective in both temperate and moderately hot ambient conditions, but they did so without experiencing negative health outcomes (e.g., dehydration, heat illness). Future research should endeavour to determine whether this remains true in even hotter ambient conditions, or when individual tasks are performed over more prolonged durations. Further, assessing which individual characteristics are important in moderating the performance response of firefighters in the heat may allow for the implementation of tailored workplace strategies.

## References

[1] Aisbett B, Wolkow A, Sprajcer M, Ferguson S (2012) "Awake, smoky, and hot": providing an evidence-base for managing the risks associated with occupational stressors encountered by wildland firefighters. Applied Ergonomics 43: 916–925. 10.1016/j.apergo.2011.12.013 22264875

[2] Rodríguez-Marroyo J, Villa J, López-Satue J, Pernía R, Carballo B, García-López J, et al (2011) Physical and thermal strain of firefighters according to the firefighting tactics used to suppress wildfires. Ergonomics 54: 1101–1108. 10.1080/00140139.2011.611895 22026953

[3] Cater H, Clancy D, Duffy K, Holgate A, Wilison B, Wood J. Fatigue on the fireground: The DPI Experience; 2007; Hobart, Australia.

[4] Cuddy J, Gaskill S, Sharkey B, Harger S, Ruby B (2007) Supplemental feedings increase self-selected work output during wildfire suppression. Medicine & Science in Sports & Exercise 39: 1004–1012. 17545892 10.1249/mss.0b013e318040b2fb

[5] Raines J, Snow R, Petersen A, Harvey J, Nichols D, Aisbett B (2012) Pre-shift fluid intake: effect on physiology, work and drinking during emergency wildfire fighting. Applied Ergonomics 43: 532–540. 10.1016/j.apergo.2011.08.007 21906723

[6] Phillips M, Payne W, Lord C, Netto K, Nichols D, Aisbett B (2012) Identification of physically demanding tasks performed during bushfire suppression by Australian rural firefighters. Applied Ergonomics 43: 435–441. 10.1016/j.apergo.2011.06.018 21802652

[7] Aisbett B, Nichols D (2007) Fighting fatigue whilst fighting bushfire: an overview of factors contributing to firefighter fatigue during bushfire suppression. The Australian Journal of Emergency Management 22: 31–39.

[8] Raines J, Snow R, Petersen A, Harvey J, Nichols D, Aisbett B (2013) The effect of prescribed fluid consumption on physiology and work behavior of wildfire fighters. Applied Ergonomics 44: 404–413. 10.1016/j.apergo.2012.10.002 23149116

[9] Bates G, Schneider J (2008) Hydration status and physiological workload of UAE construction workers: A prospective longitudinal observational study. Journal of Occupational Medicine & Toxicology 3: 1–10. 18799015 10.1186/1745-6673-3-21 PMC2561022

[10] Garrett A, Creasy R, Rehrer N, Patterson M, Cotter J (2012) Effectiveness of short-term heat acclimation for highly trained athletes. European Journal of Applied Physiology 112: 1827–1837. 10.1007/s00421-011-2153-3 21915701

[11] Garrett A, Goosens N, Rehrer N, Patterson M, Cotter J (2009) Induction and decay of short-term heat acclimation. European Journal of Applied Physiology 107: 659–670. 10.1007/s00421-009-1182-7 19727796

[12] Castle P, Mackenzie R, Maxwell N, Webborn A, Watt P (2011) Heat acclimation improves intermittent sprinting in the heat but additional pre-cooling offers no further ergogenic effect. Journal of Sports Sciences 29: 1125–1134. 10.1080/02640414.2011.583673 21777052

[13] Snook S, Ciriello V (1974) The effects of heat stress on manual handling tasks. American Industrial Hygiene Association Journal 35: 681–685. 4429074 10.1080/0002889748507088

[14] McLellan T, Jacobs I, Bain J (1993) Influence of temperature and metabolic rate on work performance with Canadian Forces NBC clothing. Aviation, Space, And Environmental Medicine 64: 587–594. 8357310

[15] McLennan J, Birch A (2005) A potential crisis in wildfire emergency response capability? Australia's volunteer firefighters. Environmental Hazards 6: 101–107.

[16] Haskell W, Lee I, Pate R, Powell K, Blair S, Franklin B, et al (2007) Physical activity and public health: Updated recommendation for adults from the American College of Sports Medicine and the American Heart Association. Circulation: 1081–1093. 17671237 10.1161/CIRCULATIONAHA.107.185649

[17] Borg G (1998) Borg's perceived exertion and pain scales: Human kinetics.

[18] Young A, Sawka M, Epstein Y, DeCristofano B, Pandolf K (1987) Cooling different body surfaces during upper and lower body exercise. Journal of Applied Physiology 63: 1218–1223. 3654466 10.1152/jappl.1987.63.3.1218

[19] Lee S, Williams W, Schneider S (2000) Core temperature measurement during submaximal exercise: Esophageal, rectal, and intestinal temperatures. National Aeronautics and Space Administration. 11001349

[20] Phillips M, Netto K, Payne W, Nichols D, Lord C, Brooksbank N, et al (2011) Frequency, intensity and duration of physical tasks performed by Australian rural firefighters during bushfire suppression In: Thornton RP, editor. Bushfire CRC & AFAC Conference. Sydney, Australia pp. 205–213.

[21] Budd G, Brotherhood J, Hendrie A, Jeffery S, Beasley F, Costin B, et al (1997) Project Aquarius 1. Stress, strain, and productivity in men suppressing Australian summer bushfires with hand tools: background, objectives, and methods. International Journal of Wildland Fire 7: 69–76.

[22] Gellish R, Goslin B, Olson R, McDonald A, Russi G, Moudgil V (2007) Longitudinal modeling of the relationship between age and maximal heart rate. Medicine & Science in Sports & Exercise 39: 822–829. 17468581 10.1097/mss.0b013e31803349c6

[23] Payne W, Portier B, Fairweather I, Zhou S, Snow R (1994) Thermoregulatory response to wearing encapsulated protective clothing during simulated work in various thermal environments. American Industrial Hygiene Association Journal 55: 529–536. 8017293 10.1080/15428119491018808

[24] Ramanathan N (1964) A new weighting system for mean surface temperature of the human body. Journal of Applied Physiology 19: 531–533. 14173555 10.1152/jappl.1964.19.3.531

[25] Field A (2007) Discovering statistics using SPSS (3rd ed). Thousand Oaks, CA: Sage Publications.

[26] Westfall P, Henning K (2013) Understanding advanced statistical methods. Florida: CRC Press.

[27] Rabe-Hesketh S, Skrondal A, Pickles A (2002) Reliable estimation of generalized linear mixed models using adaptive quadrature. The Stata Journal 2: 1–21.

[28] Rabe-Hesketh S, Skrondal A, Pickles A (2005) Maximum likelihood estimation of limited and discrete dependent variable models with nested random effects. Journal of Econometrics 128: 301–323.

[29] Molenberghs G, Verbeke G (2001) A review on linear mixed models for longitudinal data, possibly subject to dropout. Statistical Modelling 1: 235–269.

[30] McCulloch C (2003) Generalized linear mixed models: Institute of Mathematical Statistics. 1–84 p.

[31] Van Dongen H, Olofsen E, Dinges D, Maislin G (2004) Mixed-model regression analysis and dealing with interindividual differences. Essential Numerical Computer Methods: 225. 10.1016/S0076-6879(04)84010-2 15081686

[32] Singer J (1998) Using SAS PROC MIXED to fit multilevel models, hierarchical models, and individual growth models. Journal of Educational and Behavioral Statistics 23: 323–355.

[33] Tasca G, Gallop R (2009) Multilevel modeling of longitudinal data for psychotherapy researchers: I. The basics. Psychotherapy Research 19: 429–437. 10.1080/10503300802641444 19235088

[34] Cnaan A, Laird N, Slasor P (1997) Tutorial in biostatistics: Using the general linear mixed model to analyse unbalanced repeated measures and longitudinal data. Statistics in Medicine 16: 2349–2380. 9351170 10.1002/(sici)1097-0258(19971030)16:20<2349::aid-sim667>3.0.co;2-e

[35] Kachman S. An introduction to generalized linear mixed models; 2000; Athens. pp. 59–73.

[36] Burnham K, Anderson D (2002) Model selection and multimodel inference: a practical information-theoretic approach: Springer.

[37] Cheung S, McLellan T, Tenaglia S (2000) The thermophysiology of uncompensable heat stress: physiological manipulations and individual characteristics. Sports Medicine 29: 329–359. 10840867 10.2165/00007256-200029050-00004

[38] Hargreaves M (2008) Physiological limits to exercise performance in the heat. Journal of Science and Medicine in Sport 11: 66–71. 17720623 10.1016/j.jsams.2007.07.002

[39] Teague B, McLeoud R, Pascoe S (2010) 2009 Victorian Bushfires Royal Commission. Parliament of Victoria.

[40] DeCarlo L (1997) On the meaning and use of kurtosis. Psychological Methods 2: 292.

[41] Neuhaus J, McCulloch C, Boylan R (2013) Estimation of covariate effects in generalized linear mixed models with a misspecified distribution of random intercepts and slopes. Statistics in Medicine 32: 2419–2429. 10.1002/sim.5682 23203817

[42] Kenefick R, Cheuvront S (2012) Hydration for recreational sport and physical activity. Nutrition Reviews 70: 137–142. 10.1111/j.1753-4887.2012.00523.x 23121349

[43] Sawka M, Burke L, Eichner E, Maughan R, Montain S, Stachenfeld N (2007) American College of Sports Medicine position stand: Exercise and fluid replacement. Medicine & Science in Sports & Exercise 39: 377–390. 17277604 10.1249/mss.0b013e31802ca597

[44] Montain S, Coyle E (1992) Influence of graded dehydration on hyperthermia and cardiovascular drift during exercise. Journal of Applied Physiology 73: 1340–1350. 1447078 10.1152/jappl.1992.73.4.1340

[45] McGregor S, Nicholas C, Lakomy H, Williams C (1999) The influence of intermittent high-intensity shuttle running and fluid ingestion on the performance of a soccer skill. Journal of Sports Sciences 17: 895–903. 10585169 10.1080/026404199365452

[46] Hamilton M, Gonzalez-Alonso J, Montain S, Coyle E (1991) Fluid replacement and glucose infusion during exercise prevent cardiovascular drift. Journal of Applied Physiology 71: 871–877. 1757323 10.1152/jappl.1991.71.3.871

[47] Doherty M, Smith P (2005) Effects of caffeine ingestion on rating of perceived exertion during and after exercise: a meta-analysis. Scandinavian Journal of Medicine & Science in Sports 15: 69–78. 15773860 10.1111/j.1600-0838.2005.00445.x

[48] Burke L (2008) Caffeine and sports performance. Applied Physiology, Nutrition, & Metabolism 33: 1319–1334. 10.1139/H08-130 19088794

[49] Budd G, Brotherhood J, Hendrie A, Jeffery S, Beasley F, Costin B, et al (1997) Project Aquarius 6. Heat load from exertion, weather, and fire in men suppressing wildland fires. International Journal of Wildland Fire 7: 119–131.

